# Fermented NaDES–Ginger Extract Attenuates Hyperglycemia-Driven Inflammation and Endothelial Adhesion in Colorectal Cancer Through the NF-κB/COX-2 Axis

**DOI:** 10.3390/life16060927

**Published:** 2026-06-01

**Authors:** Kuen-Lin Wu, Shun-Fu Chang, Cheng-Nan Chen, Ko-Chao Lee

**Affiliations:** 1 Division of Colorectal Surgery, Department of Surgery, Kaohsiung Chang Gung Memorial Hospital, Kaohsiung 833, Taiwan; focus913@gmail.com; 2 Department of Medical Research and Development, Chang Gung Memorial Hospital Chiayi Branch, Chiayi 613, Taiwan; sfchang@cgmh.org.tw; 3 Department of Biochemical Science and Technology, National Chiayi University, Chiayi 600, Taiwan

**Keywords:** colorectal cancer, hyperglycemia, fermented ginger extract, natural deep eutectic solvent, inflammation, NF-κB, cell adhesion

## Abstract

Hyperglycemia aggravates colorectal cancer (CRC) progression by driving inflammatory pathways and facilitating tumor–endothelial cell adhesion. Ginger extract exhibits well-known anti-inflammatory properties; however, its pharmacological efficacy under such metabolic stress conditions remains largely unclear. In this study, the effects of a fermented ginger extract prepared using natural deep eutectic solvents (FNGE) were examined on high-glucose-induced pathogenesis in human CRC cells. DLD-1 cells were exposed to high-glucose conditions with or without FNGE treatment. The expression of inflammatory mediators (cyclooxygenase-2 [COX-2], prostaglandin E2 [PGE2], interleukin [*IL-6*], and *IL-8*), the adhesion capacity of CRC cells to human umbilical vein endothelial cells (HUVECs), and activation of the NF-κB signaling pathway were examined. FNGE treatment significantly and dose-dependently attenuated the high-glucose-induced upregulation of COX-2 mRNA and PGE2 secretion, while concurrently suppressing the expression of *IL-6* and *IL-8*. Furthermore, FNGE pretreatment markedly impaired CRC cell adhesion to HUVECs. Mechanistic analyses, including targeted COX-2 knockdown and pharmacological inhibition, revealed that FNGE exerts its anti-inflammatory and anti-adhesive effects primarily through the suppression of NF-κB activation, a master transcriptional regulator of these pathogenic pathways. These in vitro findings demonstrated that FNGE effectively mitigates high glucose-mediated inflammation and endothelial adhesion in CRC cells by downregulating the NF-κB/COX-2 signaling axis. Thus, this study provides a preliminary biochemical basis for the potential application of green-extracted phytochemicals under metabolic stress, highlighting the need for future in vivo validation to confirm their translational relevance.

## 1. Introduction

Colorectal cancer (CRC) ranks among the most prevalent and lethal cancers globally, consistently representing a leading cause of cancer-associated mortality across numerous countries [1]. Despite substantial advancements in cancer diagnosis and therapeutic approaches, persistent recurrence and metastasis continue to pose significant clinical challenges, often potentially compromising the overall efficacy of existing treatment modalities [2]. CRC arises from a complex interplay of several etiologies, encompassing intrinsic genetic alterations and extrinsic influences, including dietary habits, chronic inflammation, and metabolic dysregulation, all of which contribute significantly to tumor initiation and progression [3]. Hyperglycemia, a hallmark of diabetes mellitus, has been recognized as a critical risk factor in CRC pathogenesis, largely because of its capacity to induce a tumor-supportive microenvironment characterized by enhanced inflammation, angiogenesis, and oxidative stress [4].

Hyperglycemia contributes to tumorigenesis through several interconnected mechanisms. It enhances the formation of advanced glycation end products (AGEs), which bind to their cellular receptor RAGE, thereby activating downstream inflammatory pathways, notably those involving nuclear factor kappa B (NF-κB). This activation leads to the transcription of genes involved in inflammation, proliferation, and survival [5]. In CRC, enhanced expression of cyclooxygenase-2 (COX-2) and prostaglandin E2 (PGE2), with expression levels correlating with disease severity, invasion depth, and poor prognosis, has been well documented [6]. Likewise, elevated expression of *IL-6* and *IL-8* has also been reported, both of which are regulated by NF-κB and promote tumor growth, angiogenesis, and resistance to apoptosis [7]. Persistent activation of these inflammatory mediators creates a self-reinforcing loop that sustains tumor-promoting conditions.

In the metastatic cascade, the adhesion of cancer cells to vascular endothelial cells is a critical step [8]. This adhesion facilitates the extravasation and colonization of tumor cells in distant organs. High glucose levels are known to increase the expression of adhesion molecules, thereby promoting the attachment of circulating tumor cells to the endothelium and enhancing metastatic potential [9]. Disrupting these adhesion-facilitating events represents a promising therapeutic strategy to limit cancer dissemination.

Natural agents, particularly those derived from traditional medicinal plants, have gained increasing interest as complementary or alternative therapeutic agents in oncology [10]. Ginger (*Zingiber officinale*), a widely consumed spice with a long history in traditional medicine, exhibits diverse bioactivities, including robust antioxidant, anti-inflammatory, and anticancer properties [11]. The scientific literature contains numerous reports elucidating these medicinal properties and attributing them to ginger’s rich composition of polyphenols, flavonoids, and characteristic pungent principles known as gingeroids. Extensive primary studies have highlighted the potent anticancer potential of aqueous and organic ginger extracts against CRC. Specifically, active phenolic compounds such as 6-, 8-, and 10-gingerol, alongside shogaols and paradols, have been extensively documented to scavenge reactive oxygen species, modulate inflammatory cascades (e.g., NF-κB and MAPK), inhibit tumor cell proliferation, and induce apoptosis in CRC cell models [12,13,14]. The efficacy of these extracts, however, is highly dependent on the method of preparation, which directly influences the stability, structural integrity, and concentration of the enriched bioactive metabolites. Furthermore, although the baseline anti-inflammatory properties of these phytochemicals are well established, their specific pharmacological efficacy under the severe metabolic stress conditions of a hyperglycemic tumor microenvironment remains largely unexplored.

Natural deep eutectic solvents (NaDESs) have recently emerged, through advances in green chemistry, as sustainable alternatives to conventional organic solvents for plant extraction [15]. NaDES comprises naturally occurring metabolites such as betaine, lactic acid, and sugars and offers high extraction efficiency, low toxicity, and environmental compatibility [16]. NaDES can preserve the functional integrity of sensitive phytochemicals while also enhancing their solubility and bioavailability. Likewise, fermentation, a process long used in food science, can biotransform phytochemicals into more bioactive or bioavailable forms [17]. Microbially fermented ginger, particularly using *Lactobacillus* species, increases antioxidant activity and modulates the phytochemical profile—such as facilitating the biotransformation of gingerols into the highly potent 6-shogaol—toward enhanced therapeutic potential [18].

In our previous studies, we developed a fermented NaDES–ginger extract (FNGE) and demonstrated its selective cytotoxicity against drug-resistant CRC cells, concurrently sparing normal colonic mucosa [18]. Building upon these findings, the current study explores the functional impact of FNGE on key pathogenic processes in CRC under hyperglycemic stress. Specifically, we examined its effects on inflammatory mediator production, activation of NF-κB, and the adhesion of CRC cells to endothelial cells. We examined whether FNGE can attenuate inflammation-driven CRC progression to provide mechanistic insight into its potential under diabetic-like conditions. Although the NF-κB/COX-2 axis is a well-known inflammatory pathway in generalized CRC, its specific hyperactivation driven by sustained hyperglycemia remains a critical clinical challenge. Therefore, the novelty of this study lies in the use of a green-extraction biotechnology platform (NaDES coupled with fermentation) to specifically intercept this metabolism-driven inflammatory adhesion process.

## 2. Materials and Methods

### 2.1. Materials

Dulbecco’s modified Eagle medium (DMEM), fetal bovine serum (FBS), antibiotics, and other cell culture reagents were purchased from Gibco (Grand Island, NY, USA). Ginger powder was obtained from Kemyth Biotech Co., Ltd. (Taipei City, Taiwan). Pyrrolidine dithiocarbamate (PDTC), betaine, D,L-lactic acid, and other analytical-grade chemicals were purchased from Sigma-Aldrich (St. Louis, MO, USA).

### 2.2. Preparation of Fermented NaDES–Ginger Extract (FNGE)

The NaDES utilized in this study was prepared following our previous methodology [18]. Betaine and D,L-lactic acid were combined in a 1:2 molar ratio and heated at 60 °C for 1 h, followed by the addition of deionized water in a final molar ratio of 1:2:2.5 (betaine–lactic acid–water). Ginger powder was mixed with the NaDES in a solid-to-liquid ratio of 1:15, and ultrasonic-assisted extraction was performed at 200 W for 15 min. The resulting extract was filtered and centrifuged. Prior to fermentation, the pH was adjusted to 6.0. The extract was then inoculated with a selected probiotic strain, *Lactobacillus reuteri*, procured from the culture collection of National Chiayi University (Chiayi, Taiwan) [18]. The initial inoculum size was approximately 5% *v*/*v*, and the mixture was incubated at 37 °C under anaerobic conditions for 48 h. Following fermentation, the viable bacterial count reached approximately 1 × 10^9^ CFU/mL, and the final pH naturally decreased to approximately 3.8–4.2. The FNGE was filtered (0.22 μm) to remove bacterial cells, aliquoted, and stored at −20 °C in the dark. To ensure experimental consistency and eliminate batch-to-batch variation, a single pooled batch of FNGE was utilized for all functional and mechanistic assays in this study. The optimization and specific advantages of this FNGE formulation, including its superiority in phytochemical extraction and biotransformation compared with non-fermented ginger extracts and conventional solvent extracts, were comprehensively established in detail in our foundational study [18]. Furthermore, to absolutely rule out solvent interference in the current functional assays, NaDES alone (without ginger extraction) was similarly diluted in the culture medium and utilized as a vehicle control. Our control assessments confirmed that this diluted NaDES vehicle neither exhibited baseline biological activity nor affected cellular viability.

### 2.3. Cell Culture

The human CRC cell line DLD-1 was provided by the Taiwan Food Industry Research and Development Institute and maintained in DMEM supplemented with 10% FBS at 37 °C in a 5% CO_2_ incubator. Normoglycemic and hyperglycemic microenvironments were simulated in vitro by culturing cells in media containing either 5.5 mM or 25 mM D-glucose, respectively. The 5.5 mM concentration reflects standard physiological fasting blood glucose levels. The 25 mM concentration was selected to mimic severe clinical hyperglycemia characteristic of poorly controlled diabetes mellitus. This elevated glucose concentration is a widely accepted and rigorously validated in vitro model for inducing metabolic stress, hyperactivating inflammatory pathways, and investigating the intersection between metabolic disorders and cancer progression [19].

### 2.4. Cell Viability Assay

The viability of DLD-1 cells was assessed using the MTT assay. Cells were seeded at a density of 5 × 10^3^ cells/well in 96-well plates and treated with FNGE for 24 h. Subsequently, MTT (0.5 mg/mL) was added and incubated for 4 h. The resulting formazan crystals were dissolved in DMSO, and the absorbance was measured at 570 nm using a spectrophotometer [20].

### 2.5. Cell Adhesion Assay

The adhesion of DLD-1 cells to human umbilical vein endothelial cells (HUVECs) was evaluated through a standardized colorimetric assay [21]. Briefly, DLD-1 cells were cultured overnight in medium with serum at low concentrations and subsequently stimulated using high glucose. The cells (2 × 10^5^ cells/mL) were then co-cultured with HUVECs, after which non-adherent cells were removed by washing gently with phosphate-buffered saline. The remaining adherent cells were fixed with 1.0% glutaraldehyde and stained with 0.1% crystal violet for 15 min. The excess dye was removed by washing using sterile water, and the stained cells were solubilized overnight in 0.1% Triton X-100. Cell adhesion was quantified by measuring absorbance at 595 nm on a spectrophotometer. The absorbance was normalized by subtracting background absorbance obtained from crystal violet-stained HUVECs cultured alone.

### 2.6. Real-Time Quantitative Polymerase Chain Reaction (PCR)

Total RNA was extracted using TRIzol reagent, and cDNA was synthesized from 1 μg RNA. Gene expression was analyzed using SYBR Green-based qRT-PCR with specific primers for *COX-2* (forward: 5′-CAC TAC ATC CTG ACC CAC TT-3′, reverse: 5′-ATG CTC CTG CTT GAG TAT GT-3′), *IL-6* (forward: 5′-ACT CAC CTC TTC AGA ACG AAT TG-3′, reverse: 5′-CCA TCT TTG GAA GGT TCA GGT TG-3′), *IL-8* (forward: 5′-ACT GAG AGT GAT TGA GAG TGG AC-3′, reverse: 5′-AAC CCT CTG CAC CCA GTT TTC-3′), and *GAPDH* (forward: 5′-GAA GGT GAA GGT CGG AGT-3′, reverse: 5′-GAA GAT GGT GAT GGG ATT TC-3′). Expression levels were calculated using the 2^−ΔΔCt^ method. Each experiment was performed in triplicate [22].

### 2.7. siRNA Transfection

Specific and control siRNAs were synthesized by Santa Cruz Biotechnology (Dallas, TX, USA). DLD-1 cells were transfected with 200 nM siRNA using Lipofectamine RNAiMAX (Thermo Fisher Scientific, Waltham, MA, USA) according to the manufacturer’s instructions. The efficiency of transfection was verified by mRNA and protein analyses [18].

### 2.8. Nuclear Factor-κB p65 Transcription Factor Activity Assay

Nuclear extracts from the cells were prepared using a commercial kit (Cayman Chemical, Ann Arbor, MI, USA). NF-κB p65 activity was measured using an ELISA-based transcription factor assay as previously described [21], and absorbance was measured at 450 nm.

### 2.9. Statistical Analysis

All quantitative data are expressed as the mean ± standard error of the mean (SEM). Reproducibility was rigorously ensured by conducting all experiments independently at least three times to constitute independent biological replicates (n ≥ 3), using distinct cell passages. Within each independent biological replicate, quantitative assessments (including RT-qPCR, ELISA, and cell adhesion assays) were performed in technical triplicates. Statistical evaluations were conducted using SigmaPlot software (version 10.0; Systat Software, San Jose, CA, USA). For comparisons involving more than two groups, one-way analysis of variance (ANOVA) followed by Tukey’s post hoc test was used. To maximize data transparency and visually represent effect sizes and variance, quantitative bar graphs are presented with overlaid individual data points. A *p*-value < 0.05 was considered statistically significant.

## 3. Results

### 3.1. Fermented NaDES–Ginger Extract Attenuates High-Glucose-Induced COX-2 Expression and PGE2 Production

**Figure 1:**
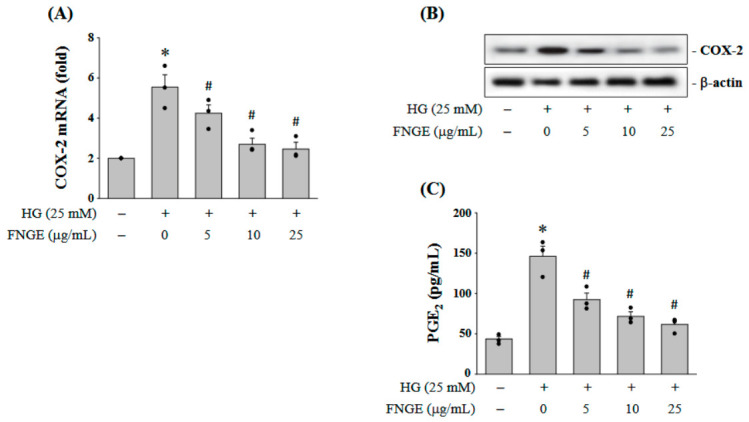
(**A**) Relative expression levels of *COX-2* mRNA in DLD-1 cells treated with high glucose (HG) and increasing concentrations of fermented NaDES–ginger extract (FNGE). (**B**) Representative Western blot analysis showing the suppression of COX-2 protein expression by FNGE under HG conditions in a dose-dependent manner. (**C**) Quantification of prostaglandin E2 (PGE2) secretion in DLD-1 cells following HG and FNGE treatment. FNGE inhibited PGE2 production in a concentration-dependent manner. Data are presented as mean ± SEM from three independent experiments. * *p* < 0.05 compared to control cells; # *p* < 0.05 compared to the HG-only group.

To ensure that the observed anti-inflammatory effects were not merely secondary to subtle cytostatic stress or cell death, the cytotoxicity of FNGE on DLD-1 cells was first evaluated using an MTT assay. The results confirmed that FNGE treatment at concentrations ranging from 5 to 25 μg/mL exerted no significant cytotoxic effects (Supplementary Figure S1A). Morphological observations confirmed that cells cultured within this concentration range exhibited healthy, adherent growth without any signs of cellular stress (Supplementary Figure S1B). Having established and justified this non-toxic working range, we subsequently found that FNGE treatment significantly and dose-dependently attenuated high-glucose-induced COX-2 mRNA and protein expression in DLD-1 cells. Compared with the high glucose-stimulated group, co-treatment with FNGE (5–25 μg/mL) resulted in a marked decrease in the *COX-2* transcript as well as protein levels (Figure 1A,B). Concurrently, the secretion of PGE2 into the culture medium was reduced significantly following FNGE treatment across the same concentration range (Figure 1C). These results demonstrate that FNGE effectively counteracts hyperglycemia-driven activation of the COX-2/PGE2 axis, highlighting its anti-inflammatory capacity in CRC cell models.

### 3.2. Fermented NaDES–Ginger Extract Downregulates IL-6 and IL-8 Expression in Colorectal Cancer Cells Exposed to High Glucose

**Figure 2:**
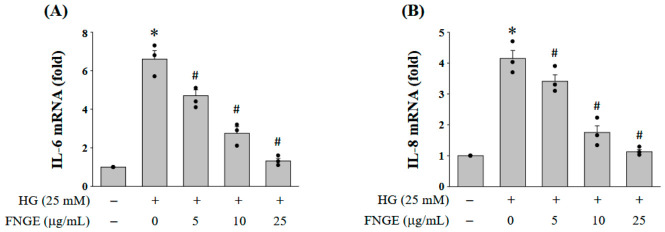
Expression levels of (**A**) interleukin-6 (*IL-6*) and (**B**) *IL-8* mRNA in DLD-1 cells treated with high glucose and various concentrations of FNGE. High glucose significantly increased the transcript levels of *IL-6* and *IL-8*, which were dose-dependently suppressed by FNGE. Data are presented as fold change relative to normoglycemic control. * *p* < 0.05 compared to control cells; # *p* < 0.05 compared to the HG-only group.

Stimulation through high glucose markedly upregulated the transcription of pro-inflammatory cytokines, elevating the mRNA levels of *IL-6* (Figure 2A) and *IL-8* (Figure 2B) in DLD-1 cells by 6.6-fold and 4.1-fold, respectively, compared with normoglycemic controls. Treatment with FNGE (5–25 μg/mL) suppressed these transcriptional surges in a dose-dependent manner. Notably, at the highest dose tested (25 μg/mL), FNGE nearly restored both cytokine expression levels to baseline. These findings further validate the efficacy of FNGE in mitigating hyperglycemia-driven inflammatory responses.

### 3.3. Fermented NaDES–Ginger Extract Inhibits Colorectal Cancer Cell Adhesion to Endothelial Cells Under Hyperglycemic Conditions

**Figure 3:**
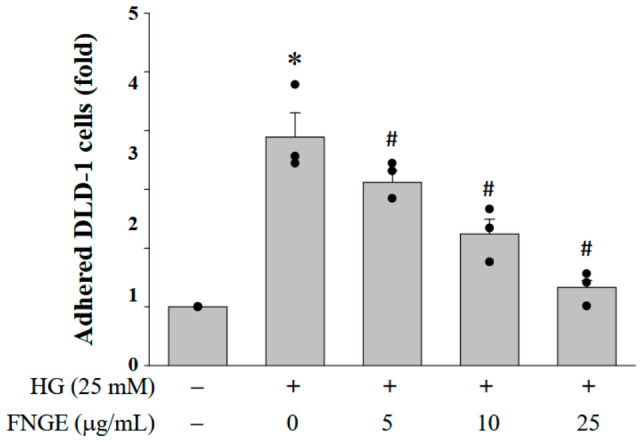
Quantification of DLD-1 cell adhesion to human umbilical vein endothelial cells (HUVECs) after exposure to high glucose. High glucose (HG) treatment significantly enhanced adhesion. FNGE pre-treatment reduced HG-induced adhesion in a dose-dependent manner. Results are representative of three independent experiments. * *p* < 0.05 compared to control cells; # *p* < 0.05 compared to the HG-only group.

The functional impact of FNGE on metastatic potential was evaluated by assessing the adhesion capacity of DLD-1 cells to HUVEC monolayers. As illustrated in Figure 3, exposure to high glucose significantly augmented the adhesion of DLD-1 cells. However, pretreatment with FNGE abrogated this high-glucose-induced adhesive behavior effectively and dose-dependently. This suggests that FNGE disrupts crucial CRC–endothelial cell interactions, thereby potentially impeding the early stages of metastasis under hyperglycemic conditions.

### 3.4. Silencing of COX-2 Suppresses PGE2 Production and Attenuates Colorectal Cancer Cell Adhesion to Human Umbilical Vein Endothelial Cells

**Figure 4:**
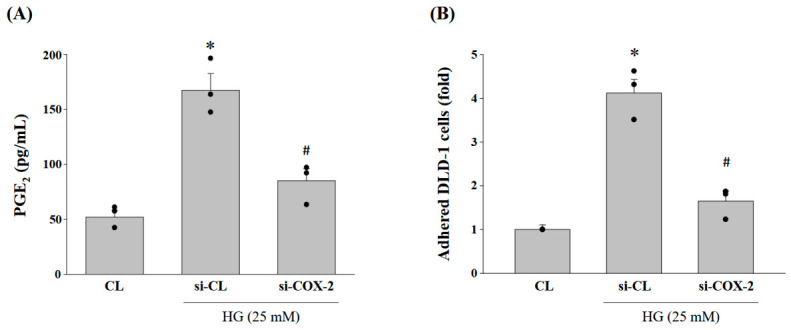
Effect of cyclooxygenase-2 (COX-2) knockdown on prostaglandin E2 (PGE2) production and DLD-1 cell adhesion to human umbilical vein endothelial cells under high glucose (HG) conditions: (**A**) Quantification of PGE2 secretion in DLD-1 cells transfected with control siRNA (si-CL) or *COX-2*-specific siRNA (si-COX-2). Targeted knockdown of COX-2 significantly suppressed HG-induced PGE2 production. (**B**) Determination of adhesion capacity of DLD-1 cells to HUVEC monolayers. Cells transfected with si-COX-2 exhibited dramatically reduced adhesion under HG stimulation compared to the control group. These results highlight the mediating role of the COX-2/PGE2 axis in HG-driven metastatic adhesion. * *p* < 0.05 compared to the control cells; # *p* < 0.05 compared to the HG-treated si-CL group.

The specific role of COX-2 in this adhesive process was delineated by transfecting DLD-1 cells with COX-2-specific siRNA. We first verified the functional efficacy of gene silencing, demonstrating that targeted COX-2 knockdown significantly suppressed high-glucose-induced PGE2 production (Figure 4A). Following this validation, subsequent adhesion assays revealed that silencing COX-2 dramatically reduced the high-glucose-induced adhesion of DLD-1 cells to HUVECs (Figure 4B). These findings firmly link the COX-2/PGE2 axis to metastatic adhesion and validate COX-2 as a critical downstream effector modulated by the anti-metastatic action of FNGE.

### 3.5. Fermented NaDES–Ginger Extract Suppresses NF-κB Activation in Colorectal Cancer Cells

**Figure 5:**
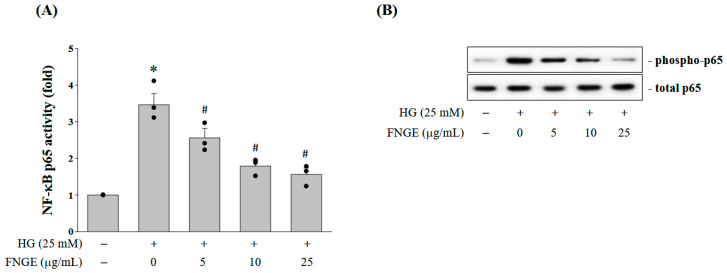
Effect of fermented NaDES–ginger extract (FNGE) on NF-κB activation in high glucose (HG)-treated DLD-1 cells: (**A**) Nuclear factor-kappa B (NF-κB) p65 activity determined by an enzyme-linked immunosorbent assay. FNGE dose-dependently inhibited HG-induced NF-κB activity. (**B**) Representative Western blot analysis demonstrating that FNGE treatment markedly suppressed the HG-induced phosphorylation of p65 (phospho-p65). Total p65 was used as an internal control. Data are presented as mean ± SEM. * *p* < 0.05 compared to control cells; # *p* < 0.05 compared to the HG-only group.

The upstream molecular mechanisms were elucidated by assessing whether FNGE targets the NF-κB pathway to exert its protective effects. Indeed, FNGE treatment significantly and dose-dependently blunted NF-κB p65 activity in high glucose-exposed DLD-1 cells (Figure 5A). To further corroborate this finding at the protein level, Western blot analysis revealed that FNGE effectively inhibited high-glucose-induced phosphorylation of the NF-κB p65 subunit (Figure 5B). This pronounced inhibitory effect aligns closely with the observed reductions in pro-inflammatory mediator expression and cellular adhesion. Taken together, these results confirm that the therapeutic efficacy of FNGE in hyperglycemia-associated CRC progression is mechanistically rooted in its ability to suppress the NF-κB signaling cascade.

### 3.6. Nuclear Factor-κB Mediates High-Glucose-Induced Inflammation and Adhesion in Colorectal Cancer Cells

**Figure 6:**
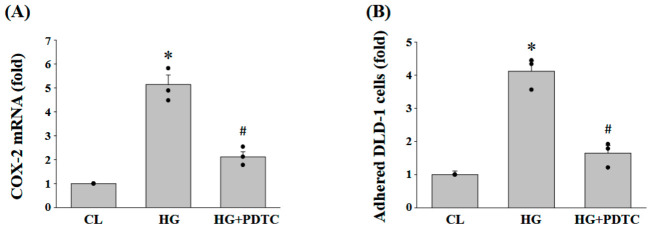
Verification of nuclear factor-kappa B (NF-κB) as the central mediator using the pharmacological inhibitor pyrrolidine dithiocarbamate (PDTC). DLD-1 cells were treated with PDTC under HG conditions: (**A**) Pharmacological inhibition of NF-κB robustly abolished HG-induced cyclooxygenase-2 (COX-2) expression. (**B**) PDTC treatment concurrently reduced HG-driven adhesion of DLD-1 cells to HUVEC monolayers. Each bar represents mean ± SEM. * *p* < 0.05 compared to the control cells; # *p* < 0.05 compared to the HG-treated group.

To conclusively verify that suppression of NF-κB is directly responsible for reversing these pathogenic phenotypes, DLD-1 cells were treated with the NF-κB inhibitor PDTC. Consistent with the effects of FNGE, pharmacological inhibition of NF-κB robustly abolished the high-glucose-induced upregulation of COX-2 expression (Figure 6A) and concurrently reduced the adhesion of DLD-1 cells to HUVECs (Figure 6B). These findings establish NF-κB as the pivotal upstream mediator driving the inflammatory and adhesive processes in hyperglycemia-stressed CRC cells, further validating it as the primary functional target of FNGE.

## 4. Discussion

CRC remains a formidable global health challenge, primarily due to its high propensity for recurrence and metastasis [23]. Hyperglycemia, a hallmark of metabolic disorders such as diabetes mellitus, has increasingly been recognized as a critical exacerbating factor in CRC progression. It establishes a pro-tumorigenic microenvironment by driving oxidative stress, amplifying inflammatory signaling, and facilitating pathogenic cancer cell–endothelial cell interactions [19]. In the current study, we provide compelling evidence that a fermented natural deep eutectic solvent-ginger extract (FNGE)—prepared using a green and sustainable extraction platform—exhibits profound anti-inflammatory and anti-adhesive properties in CRC cells subjected to hyperglycemic stress. These findings significantly advance our understanding of the therapeutic mechanisms of FNGE, supporting its future development as a complementary anticancer modality.

Chronic inflammation is a hallmark of the hyperglycemia-driven tumor microenvironment. Our study demonstrates that FNGE treatment significantly suppresses the robust upregulation of the COX-2/PGE2 signaling axis in high glucose-stimulated DLD-1 cells. COX-2 overexpression in colorectal tumors is clinically well documented and is deeply implicated in driving tumor progression through the promotion of cellular proliferation, evasion of apoptosis, and induction of angiogenesis [24]. Importantly, our data reveal that FNGE blunts this axis at both the transcriptional (COX-2 mRNA) and translational (COX-2 protein) levels, consequently inhibiting the synthesis of its downstream lipid mediator, PGE2. This dose-dependent inhibition aligns with previous studies suggesting that ginger-derived bioactive compounds, such as 6-shogaols and related phenolic compounds, act as potent modulators of inflammatory enzymes [25]. Beyond the COX-2 pathway, FNGE also markedly attenuated the transcription of two pivotal pro-inflammatory cytokines, *IL-6* and *IL-8*. Given that *IL-6* promotes cancer cell survival and immune evasion [26], while *IL-8* orchestrates local angiogenic and chemotactic responses [27], the ability of FNGE to concurrently suppress these mediators underscores its broad capacity to disrupt the autocrine and paracrine inflammatory loops that sustain tumor aggressiveness.

Equally important is the observed inhibitory effect of FNGE on CRC cell adhesion to the endothelial monolayer. Tumor cell adherence to the vascular endothelium is an obligatory early step in the metastatic cascade, facilitating the anchorage of circulating cancer cells to distant vascular beds and initiating extravasation [28]. While hyperglycemia is known to upregulate adhesion molecules and accelerate metastatic dissemination [29], our in vitro model demonstrated that FNGE pretreatment effectively abrogated the high-glucose-induced hyperadhesive behavior of DLD-1 cells toward HUVECs. Most notably, through targeted genetic silencing, we demonstrated that the ablation of COX-2 not only suppressed PGE2 production but also directly diminished cellular adhesion. Extensive literature indicates that hyperglycemia-induced activation of the COX-2/PGE2 axis directly upregulates adhesion molecules like ICAM-1 and VCAM-1 on the endothelial cell surface [30,31]. By downregulating this upstream axis, FNGE deprives the microenvironment of the signals required to sustain these adhesion molecules. This mechanistic validation highlights a previously underappreciated role for the COX-2/PGE2 axis in regulating CRC–endothelial cell interactions under metabolic stress, confirming this axis as a critical vulnerability effectively targeted by the anti-metastatic action of FNGE.

To provide a biochemical rationale for these profound anti-inflammatory and anti-adhesive effects, it is essential to consider the established phytochemical profile of our strictly standardized FNGE formulation. As comprehensively characterized via HPLC in our foundational methodology study [18], the fermentation of ginger in NaDES significantly facilitates the biotransformation of ginger metabolites, leading to a marked enrichment in total phenolic content and the highly potent bioactive compound, 6-shogaol. Extensive literature has established that 6-shogaol and other phenolic antioxidants act as exceptionally potent inhibitors of the NF-κB signaling cascade, effectively preventing its activation and subsequently downregulating COX-2 and PGE2 expression [32,33]. Furthermore, these enriched antioxidants are widely reported to mitigate oxidative stress and endothelial inflammation, which are critical drivers of pathogenesis in hyperglycemic microenvironments [34]. While our previous analysis also noted significant increases in specific, currently uncharacterized biotransformed metabolites following fermentation [18], the firmly established presence of 6-shogaol and elevated overall phenolic constituents provides a robust molecular basis for our findings. The synergistic interplay of these enriched phytochemicals directly corroborates the pharmacological suppression of hyperglycemia-induced tumor–endothelial cell adhesion observed in our DLD-1 model.

NF-κB emerged as the central transcriptional hub coordinating these pathogenic responses [5]. We observed robust phosphorylation and transcriptional activation of the NF-κB p65 subunit under high-glucose conditions, which coincided with the phenotypic increases in COX-2, *IL-6*, and *IL-8* expression, alongside enhanced cellular adhesion. FNGE treatment dose-dependently reversed these molecular events, indicating its potent role as an NF-κB repressor. The indispensable role of NF-κB was further verified using the pharmacological inhibitor PDTC, which phenocopied the protective effects of FNGE by markedly abolishing high-glucose-induced COX-2 expression and impairing CRC cell adhesion. However, PDTC is not an exclusively specific NF-κB inhibitor; as documented in the literature, it also possesses potent antioxidant and metal-chelating properties [35]. Specifically, PDTC can sequester transition metal ions, which serve as essential cofactors for various redox-sensitive enzymes and signaling molecules. Given that hyperglycemia is intrinsically linked to the generation of reactive oxygen species (ROS) and the mobilization of intracellular metal ions, these pleiotropic effects may also contribute to the observed outcomes. Consequently, PDTC may exert its protective effects through a combination of NF-κB suppression and broader modulation of metal-dependent oxidative signaling. Nevertheless, the robustness of our mechanistic claim is significantly strengthened by the fact that the anti-adhesive phenotypes observed with PDTC were closely reflected by the highly specific targeted knockdown of COX-2 using siRNA (Figure 4). This convergence of pharmacological and genetic evidence strongly suggests that, irrespective of the auxiliary effects of PDTC, suppression of the NF-κB/COX-2 axis remains a pivotal mechanism through which FNGE exerts its effects. Taken together, these mechanistic insights strongly suggest that the anti-inflammatory and anti-adhesive activities of FNGE are primarily orchestrated through profound downregulation of the NF-κB signaling cascade, thereby effectively interrupting the inflammation-metastasis link.

Although our findings clearly demonstrate the suppression of this classical NF-κB/COX-2 cascade by FNGE, the complexity of the tumor microenvironment suggests that upstream regulators are likely involved. For instance, high glucose induces the accumulation of AGEs and triggers reactive oxygen species (ROS), both of which are upstream activators of NF-κB. Future omics-based studies, including transcriptomics and targeted proteomics, are warranted to map the broader signaling networks, such as the RAGE/ROS axis and alternative MAP kinase pathways, modulated by this unique phytochemical mixture. Beyond direct suppression of the NF-κB/COX-2 axis, the therapeutic potential of ginger-derived bioactives in FNGE may involve a broader regulatory network [12]. Recent evidence suggests that natural phytochemicals from medicinal plants exhibit multifaceted anticancer properties by simultaneously modulating inflammatory signaling, oxidative stress, and apoptotic pathways [36]. Specifically, attenuation of pro-inflammatory mediators often correlates with reduced intracellular ROS levels, which may further sensitize CRC cells to apoptotic stimuli and restrict their metastatic plasticity [37]. While the current study primarily focuses on the anti-adhesive capacity of FNGE, it is highly plausible that inhibition of the COX-2/PGE2 axis contributes to a broader rebalancing of the tumor-promoting inflammatory microenvironment. Such mechanistic intersections reinforce the translational potential of FNGE as a multitargeted strategy for suppressing the aggressive progression of solid tumors, particularly under the metabolic stress of hyperglycemia.

Furthermore, although the current study focuses on pathogenic mechanisms under hyperglycemic stress, it builds upon the technological foundation established in our previous work. We previously demonstrated that the specific combination of green NaDES extraction and microbial fermentation superiorly enriches bioactive gingeroids compared with conventional non-fermented or aqueous/ethanolic extraction methods [18]. Thus, the application of this optimized, high-efficiency FNGE formulation allowed us to selectively investigate its advanced anti-inflammatory effects in the metabolic CRC model without the confounding influence of suboptimal extraction yields.

From a translational perspective, the implementation of NaDES for ginger extraction represents a highly innovative and eco-friendly paradigm. Unlike conventional volatile organic solvents, NaDES is composed of non-toxic natural metabolites that maximize extraction efficiency while preserving the structural integrity and potentially enhancing the bioavailability of sensitive phytochemicals [18]. Furthermore, microbial fermentation using *Lactobacillus reuteri* biotransforms and enriches the extract’s pharmacological profile. As previously reported, such microbial biotransformation can generate secondary metabolites with superior bioactivity and improved absorption kinetics [38].

To fully realize the clinical translational potential of these natural bioactives, future studies should explore advanced formulation strategies. As highlighted in recent literature, integrating bioactive phytochemicals with nanoformulation-assisted delivery systems or combinational approaches can significantly overcome intrinsic bioavailability barriers and maximize therapeutic performance in cancer therapy [39]. Developing FNGE into such specialized delivery platforms could facilitate targeted release of gingeroids within the colon, thereby maximizing anti-inflammatory efficacy while minimizing systemic fluctuations.

Although the robust anti-inflammatory and anti-adhesive effects have been demonstrated herein, this study has certain limitations that warrant consideration. First, the in vitro mechanistic evaluations were primarily conducted using a single CRC cell line (DLD-1). Colorectal tumors are highly heterogeneous, and different cell lines possess distinct mutational landscapes (e.g., KRAS or PIK3CA mutations observed in HCT116 or SW480 cells) that may influence their metabolic plasticity and responsiveness to phytochemical interventions. Therefore, future studies should validate the generalizability of FNGE-mediated NF-κB/COX-2 suppression across a broader panel of CRC cell lines. Second, although the safety profile and lack of cytotoxicity of FNGE toward non-cancerous normal colonic epithelial cells were comprehensively established in our previous investigation [18], the current study focuses solely on cancer-endothelial cell interactions. The full translational applicability of these findings therefore requires further confirmation using complex co-culture systems or in vivo diabetic CRC animal models. Third, although our targeted knockdown and pharmacological inhibition assays clearly demonstrate that the NF-κB/COX-2 axis is functionally required for the observed phenotypes, the precise causal sequence remains to be definitively established. Future studies employing specific rescue models, such as exogenous PGE2 supplementation or constitutive p65 activation, are necessary to determine whether restoration of this pathway completely abolishes the anti-adhesive benefits of FNGE. Finally, due to practical constraints, batch-specific LC-MS/MS or HPLC phytochemical profiling was not performed in the current study; thus, the chemical foundation relies on the strictly standardized extraction protocol validated in our previous foundational work.

In summary, this in vitro study provides a robust biochemical rationale for exploring FNGE as a potential intervention for CRC, particularly for patients with metabolic comorbidities such as diabetes mellitus. By comprehensively suppressing the NF-κB/COX-2 axis, FNGE simultaneously mitigates tumor-promoting inflammation and disrupts early metastatic cell adhesion. Future in vivo investigations using complex diabetic CRC models are strongly warranted to validate these cellular findings, evaluate systemic safety, and explore the potential synergistic efficacy of FNGE in combination with standard chemotherapeutic or targeted regimens.

## 5. Conclusions

The present in vitro study demonstrates that the strictly standardized FNGE formulation effectively attenuates hyperglycemia-driven inflammatory responses and cellular adhesion in CRC cells. Grounded in our foundational methodology, the formulation’s established enrichment in 6-shogaol and phenolic antioxidants provides a robust biochemical rationale for these functional observations. We verified that FNGE dose-dependently suppresses the expression of pro-inflammatory mediators and impairs tumor–endothelial cell adhesion, primarily through the downregulation of the NF-κB/COX-2 signaling axis. Ultimately, these findings provide foundational mechanistic evidence for intercepting metabolic stress-exacerbated metastasis, highlighting the translational potential of this green-extraction platform while underscoring the necessity for future in vivo validation.

While these findings highlight the pharmacological potential of integrating green NaDES extraction with microbial fermentation, we acknowledge that the current evidence is limited to cellular models. Therefore, the results should be interpreted as foundational mechanistic evidence rather than a direct therapeutic strategy. Comprehensive in vivo investigations need to be conducted to establish the translational applicability of FNGE to evaluate its systemic bioavailability, safety profile, and efficacy across diverse genetic backgrounds. Furthermore, exploration of advanced strategies, such as nanoformulation-assisted delivery, will be essential to bridge the gap between these cellular findings and their future potential as functional interventions for metabolically driven malignancies.

## Data Availability

The data that support the findings of the current study are available from the corresponding author upon reasonable request.

## Supplementary Materials

The following supporting information can be downloaded at: https://www.mdpi.com/article/10.3390/life16060927/s1, Figure S1: Assessment of cell viability and morphology in DLD-1 cells treated with FNGE. (A) Cell viability (%) of DLD-1 cells treated with fermented NaDES–ginger extract (FNGE) at concentrations ranging from 5 to 25 μg/mL. No significant cytotoxic effects were observed compared to the control group. (B) Representative morphological observations of DLD-1 cells in the control group (CL) and those treated with 25 μg/mL of FNGE. The cells exhibited healthy, adherent growth with typical morphology, showing no signs of cellular stress. Data are presented as mean ± SEM from three independent experiments..

## References

[1] Sung H., Ferlay J., Siegel R.L., Laversanne M., Soerjomataram I., Jemal A., Bray F. (2021). Global cancer statistics 2020: GLOBOCAN estimates of incidence and mortality worldwide for 36 cancers in 185 countries. CA Cancer J. Clin..

[2] Ma S.C., Zhang J.Q., Yan T.H., Miao M.X., Cao Y.M., Cao Y.B., Zhang L.C., Li L. (2023). Novel strategies to reverse chemoresistance in colorectal cancer. Cancer Med..

[3] Malki A., ElRuz R.A., Gupta I., Allouch A., Vranic S., Al Moustafa A.E. (2020). Molecular mechanisms of colon cancer progression and metastasis: Recent insights and advancements. Int. J. Mol. Sci..

[4] Yu G.H., Li S.F., Wei R., Jiang Z. (2022). Diabetes and colorectal cancer risk: Clinical and therapeutic implications. J. Diabetes Res..

[5] Alsulami F.J., Shaheed S.U. (2024). Role of natural antioxidants in cancer. Cancer Treat. Res..

[6] Sheng J., Sun H., Yu F.B., Li B., Zhang Y., Zhu Y.T. (2020). The role of cyclooxygenase-2 in colorectal cancer. Int. J. Med. Sci..

[7] Kim B., Seo Y., Kwon J.H., Shin Y., Kim S., Park S.J., Park J.J., Cheon J.H., Kim W.H., Kim T.I. (2021). IL-6 and IL-8, secreted by myofibroblasts in the tumor microenvironment, activate HES1 to expand the cancer stem cell population in early colorectal tumor. Mol. Carcinog..

[8] Hilfenhaus G., Mompeón A., Freshman J., Prajapati D.P., Hernandez G., Freitas V.M., Ma F., Langenbacher A.D., Mirkov S., Song D. (2021). A high-content screen identifies drugs that restrict tumor cell extravasation across the endothelial barrier. Cancer Res..

[9] Sugiura K., Okabayashi K., Seishima R., Ishida T., Shigeta K., Tsuruta M., Hasegawa H., Kitagawa Y. (2022). Metformin inhibits the development and metastasis of colorectal cancer. Med. Oncol..

[10] Alzate-Yepes T., Pérez-Palacio L., Martínez E., Osorio M. (2023). Mechanisms of action of fruit and vegetable phytochemicals in colorectal cancer prevention. Molecules.

[11] Li C., Li J., Jiang F., Tzvetkov N.T., Horbanczuk J.O., Li Y., Atanasov A.G., Wang D. (2021). Vasculoprotective effects of ginger (Zingiber officinale Roscoe) and underlying molecular mechanisms. Food Funct..

[12] Xiang S., Jian Q., Chen W., Xu Q., Li J., Wang C., Wang R., Zhang D., Lin J., Zheng C. (2024). Pharmacodynamic components and mechanisms of ginger (*Zingiber officinale*) in the prevention and treatment of colorectal cancer. J. Ethnopharmacol..

[13] Hu S.M., Yao X.H., Hao Y.I., Pan A.H., Zhou X.W. (2020). 8-Gingerol regulates colorectal cancer cell proliferation and migration through the EGFR/STAT/ERK pathway. Int. J. Oncol..

[14] Chen M., Tong C., Wu Q., Zhong Z., He Q., Zeng L., Xiao L. (2023). 6-Shogaol Inhibits the Cell Migration of Colon Cancer by Suppressing the EMT Process Through the IKKβ/NF-κB/Snail Pathway. Integr. Cancer Ther..

[15] Li D. (2022). Natural deep eutectic solvents in phytonutrient extraction and other applications. Front. Plant Sci..

[16] Zuo J., Geng S., Kong Y., Ma P., Fan Z., Zhang Y., Dong A. (2023). Current progress in natural deep eutectic solvents for the extraction of active components from plants. Crit. Rev. Anal. Chem..

[17] Lee K.C., Wu K.L., Yen C.K., Chang S.F., Chen C.N., Lu Y.C. (2022). Inhibition of NLRP3 by fermented quercetin decreases resistin-induced chemoresistance to 5-fluorouracil in human colorectal cancer cells. Pharmaceuticals.

[18] Lee K.C., Wu K.L., Chang S.F., Chang H.I., Chen C.N., Chen Y.Y. (2022). Fermented ginger extract in natural deep eutectic solvent enhances cytotoxicity by inhibiting NF-κB mediated CXC chemokine receptor 4 expression in oxaliplatin-resistant human colorectal cancer cells. Antioxidants.

[19] Jeong H.S., Lee D.H., Kim S.H., Lee C.H., Shin H.M., Kim H.R., Cho C.H. (2022). Hyperglycemia-induced oxidative stress promotes tumor metastasis by upregulating vWF expression in endothelial cells through the transcription factor GATA1. Oncogene.

[20] Lee K.C., Wu K.L., Yen C.K., Chen C.N., Chang S.F., Huang W.S. (2021). 6-Shogaol antagonizes the adipocyte-conditioned medium-initiated 5-fluorouracil resistance in human colorectal cancer cells through controlling the SREBP-1 level. Life.

[21] Huang W.S., Yang J.T., Lu C.C., Chang S.F., Chen C.N., Su Y.P., Lee K.C. (2015). Fulvic acid attenuates resistin-induced adhesion of HCT-116 colorectal cancer cells to endothelial cells. Int. J. Mol. Sci..

[22] Lee K.C., Yen C.K., Chen C.N., Chang S.F., Lu Y.C., Huang W.S. (2021). Drug resistance of CPT-11 in human DLD-1 colorectal cancer cells through MutS homolog 2 upregulation. Int. J. Med. Sci..

[23] Morgan E., Arnold M., Gini A., Lorenzoni V., Cabasag C.J., Laversanne M., Vignat J., Ferlay J., Murphy N., Bray F. (2023). Global burden of colorectal cancer in 2020 and 2040: Incidence and mortality estimates from GLOBOCAN. Gut.

[24] Hidalgo-Estévez A.M., Stamatakis K., Jiménez-Martínez M., López-Pérez R., Fresno M. (2020). Cyclooxygenase-2-regulated genes: An alternative avenue to the development of new therapeutic drugs for colorectal cancer. Front. Pharmacol..

[25] Kim J.E., Park K.H., Park J., Kim B.S., Kim G.S., Hwang D.G. (2025). Immunomodulatory potential of 6-gingerol and 6-shogaol in Lactobacillus plantarum-fermented Zingiber officinale extract on murine macrophages. Int. J. Mol. Sci..

[26] Johnson D.E., O’Keefe R.A., Grandis J.R. (2018). Targeting the IL-6/JAK/STAT3 signalling axis in cancer. Nat. Rev. Clin. Oncol..

[27] Meier C., Brieger A. (2025). The role of IL-8 in cancer development and its impact on immunotherapy resistance. Eur. J. Cancer.

[28] Fares J., Fares M.Y., Khachfe H.H., Salhab H.A., Fares Y. (2020). Molecular principles of metastasis: A hallmark of cancer revisited. Signal Transduct. Target. Ther..

[29] Wang D., Wang F., Kong X., Li Q., Shi H., Zhao S., Li W., Li Y., Meng J. (2022). The role of metabolic reprogramming in cancer metastasis and potential mechanism of traditional Chinese medicine intervention. Biomed. Pharmacother..

[30] Zhang H., Dellsperger K.C., Zhang C. (2012). The link between metabolic abnormalities and endothelial dysfunction in type 2 diabetes: An update. Basic Res. Cardiol..

[31] Madonna R., Giovannelli G., Confalone P., Renna F.V., Geng Y.J., De Caterina R. (2016). High glucose-induced hyperosmolarity contributes to COX-2 expression and angiogenesis: Implications for diabetic retinopathy. Cardiovasc. Diabetol..

[32] Salama A.F., El-Far A.H., Anbar E.A., El-Naggar S.A., Elshazli R.M., Elmetwalli A. (2024). Gingerol and/or sorafenib attenuates the DAB-induced HCC and hepatic portal vein dilatation via ATG4/CASP3 and COIIV/COX-2/NF-kappaB expression. Med. Oncol..

[33] Song S., Dang M., Kumar M. (2019). Anti-inflammatory and renal protective effect of gingerol in high-fat diet/streptozotocin-induced diabetic rats via inflammatory mechanism. Inflammopharmacology.

[34] Wang R., Santos J.M., Dufour J.M., Stephens E.R., Miranda J.M., Washburn R.L., Hibler T., Kaur G., Lin D., Shen C.-L. (2022). Ginger Root Extract Improves GI Health in Diabetic Rats by Improving Intestinal Integrity and Mitochondrial Function. Nutrients.

[35] Moon S.K., Jung S.Y., Choi Y.H., Lee Y.C., Patterson C., Kim C.H. (2004). PDTC, metal chelating compound, induces G1 phase cell cycle arrest in vascular smooth muscle cells through inducing p21Cip1 expression: Involvement of p38 mitogen activated protein kinase. J. Cell. Physiol..

[36] Rana J.N., Mumtaz S. (2025). Prunin: An Emerging Anticancer Flavonoid. Int. J. Mol. Sci..

[37] Ahmed M.B., Islam S.U., Alghamdi A.A.A., Kamran M., Ahsan H., Lee Y.S. (2022). Phytochemicals as Chemo-Preventive Agents and Signaling Molecule Modulators: Current Role in Cancer Therapeutics and Inflammation. Int. J. Mol. Sci..

[38] Dissanayake I.H., Tabassum W., Alsherbiny M., Chang D., Li C.G., Bhuyan D.J. (2025). Lactic acid bacterial fermentation as a biotransformation strategy to enhance the bioavailability of phenolic antioxidants in fruits and vegetables: A comprehensive review. Food Res. Int..

[39] Rana J.N., Gul K., Mumtaz S. (2025). Isorhamnetin: Reviewing Recent Developments in Anticancer Mechanisms and Nanoformulation-Driven Delivery. Int. J. Mol. Sci..

