# Supplementary material for: Fermented NaDES–Ginger Extract Attenuates Hyperglycemia-Driven Inflammation and Endothelial Adhesion in Colorectal Cancer Through the NF-κB/COX-2 Axis
Source: Life (Basel). 2026 Jun 1;16(6):927. doi: 10.3390/life16060927 (PMC13302201; doi:10.3390/life16060927)

Figure S1

(A)

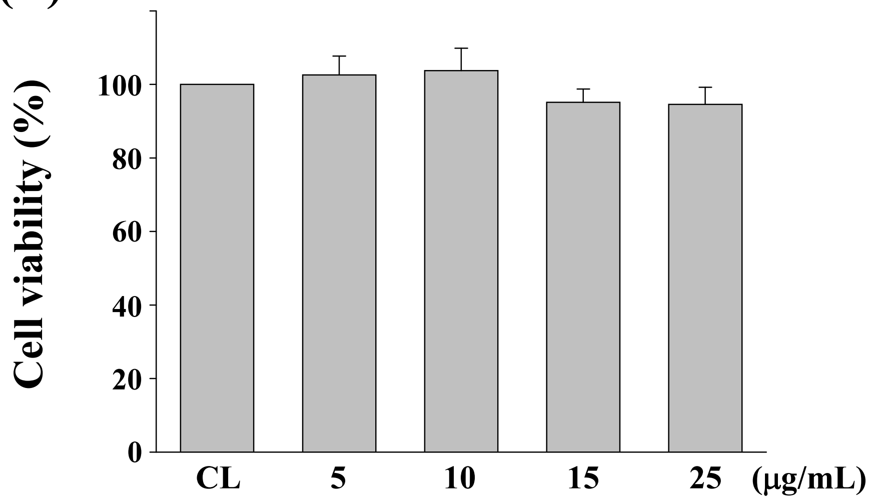

(B)

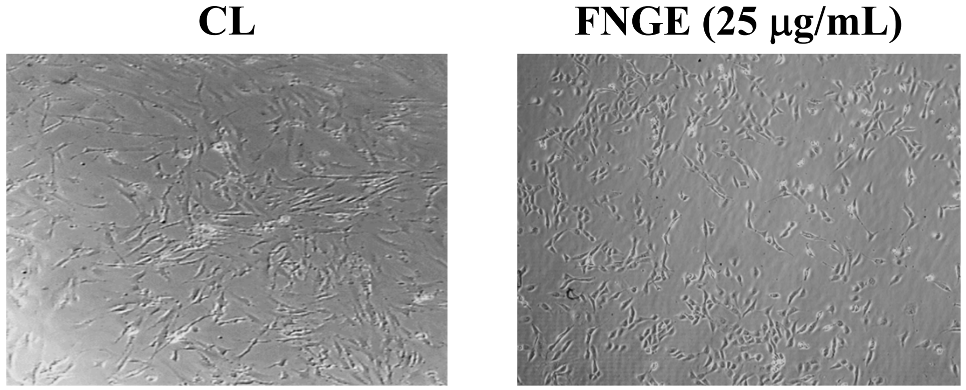

(A) FNGE treatment at concentrations ranging from 5 to 25 μg/mL exerted no significant cytotoxic effects on DLD-1 cells.

(B) Morphological observations confirmed that cells cultured within 25 μg/mL of FNGE treatment exhibited healthy, adherent growth without any signs of cellular stress.

Original, representative blots corresponding to Figures in this study.

Figure 1B

COX-2 (74 kD)

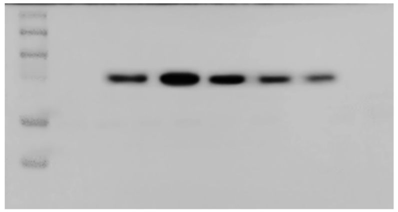

β-actin (45 kD)

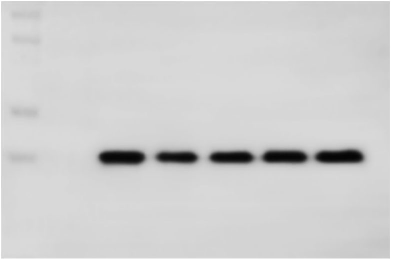

Figure 5B

- Phospho p65 (65 kD)

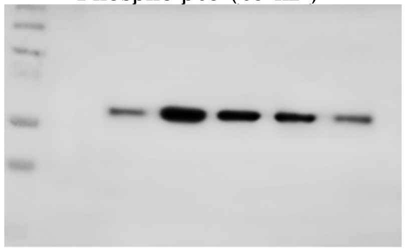

p65 (65 kD)

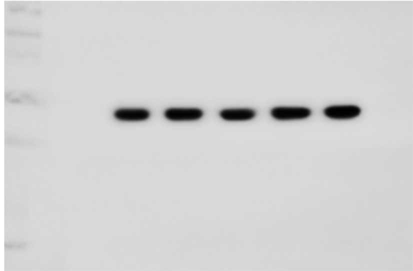

Supplement: Supplementary file 1 [file life-16-00927-s001.zip › life-4298362-supplementary.pdf]
